# Effects of a Complex Environment on Fatigue and Self-Healing Characterization of Asphalt Composites Containing Rock Asphalt

**DOI:** 10.3390/ma17102453

**Published:** 2024-05-19

**Authors:** Ruixia Li, Shangjun Yu, Hailong Chen, Jiahui Wu, Yijun Chen, Jinchao Yue

**Affiliations:** 1 School of Water Conservancy and Transportation, Zhengzhou University, Zhengzhou 450001, China; 2 Henan Central Construction Engineering Co., Ltd., Zhengzhou 450016, China

**Keywords:** rock asphalt, fine aggregate matrix (FAM), fatigue and self-healing properties, UV aging, freeze–thaw, UV aging–freeze–thaw, microstructure

## Abstract

In recent years, asphalt pavement has been subjected to varied environmental conditions during its service life, conditions that predispose it to deformation and cracking. To enhance the performance of asphalt pavement, rock asphalt has been selected as a modifier due to its good compatibility with virgin asphalt binder and its ability to improve the fatigue cracking resistance of asphalt mixtures. Although scholars have conducted some studies on rock asphalt mixtures, research on the fatigue and self-healing performance of these mixtures under conditions such as ultraviolet (UV) aging and freeze–thaw remains limited. This paper presents findings from a study that employs a combined fatigue-healing test to assess the impact of such complex environmental factors on the fatigue and self-healing properties of fine aggregate matrix (FAM) mixtures containing three types of rock asphalts, i.e., Buton, Qingchuan (QC), and Uintaite Modifier (UM). The analysis of fatigue-healing test results, grounded in viscoelastic continuum damage (VECD) theory, indicates that rock asphalt can extend the fatigue life of FAM mixtures, albeit with a concomitant decrease in their self-healing capabilities. The study further reveals that UV aging, freeze–thaw, and UV aging–freeze–thaw conditions all led to a diminution in the fatigue and self-healing properties of FAM mixtures. However, FAM mixtures containing rock asphalt demonstrated greater resilience against these reductions. Atomic force microscope (AFM) results indicate that UV aging reduced the number of bee-structures and enlarged their area, whereas the incorporation of rock asphalt enhanced the uniformity of these structures’ distribution, thereby improving the fatigue cracking resistance of FAM mixtures. Fourier transform infrared spectroscopy (FTIR) analysis reveals that while UV aging increased the carbonyl and sulfoxide indices within the asphalt binder, rock asphalt is effective in mitigating this effect to a certain degree, thereby enhancing the aging resistance of FAM mixtures.

## 1. Introduction

Rock asphalt is a naturally occurring variety of asphalt that is created through oxidation, deposition, heat, pressure, catalysts, and microorganisms [1]. Typical kinds of rock asphalt conclude three varieties, i.e., the Qingchuan (QC) rock asphalt from Sichuan, China, the Uintaite Modifier (UM) rock asphalt from Kermanshah, Iran, and the Buton rock asphalt from Buton, Indonesia [2]. Due to rock asphalt having great compatibility with asphalt binder, it is commonly chosen as a modifier of asphalt binder [3,4,5,6,7].

In recent years, researchers have studied the influence of rock asphalt on many characteristics of performance. Li and co-workers evaluated the rheological properties and low-temperature performance of fine aggregate mixtures containing rock asphalt, finding that the addition of rock asphalt enhanced the stiffness of the materials but had a slight harmful impact on low-temperature performance [1]. Zou and co-workers conducted field performance tests on asphalt concrete mixtures modified with different proportions of Buton rock asphalt, noting that the water stability and fatigue life of the modified mixtures were superior to those of unmodified mixtures [8]. Yilmaz and co-workers added rock asphalt as filler to hot-mix asphalt (HMA) mixtures and observed significantly increased stiffness, permanent deformation, and fatigue resistance of HMA [9]. Sun and co-workers prepared modified asphalt binders with concentrations of Xinjiang asphaltite ranging from 4% to 16% in the laboratory, finding that with the increase in concentration, the resistance to deformation of the asphalt binders enhanced [10]. Wang and co-workers found that the concentrations of rock asphalt affect fatigue life, with less than 20% having a growth effect and more having a reverse effect [11]. Zhou found that the fatigue resistance of the Buton rock asphalt mixture is optimal when the content of Buton rock asphalt is 7.5% [12]. Sun and co-workers undertook intermittent loading tests related to fatigue-induced healing, revealing that rock asphalt imparts an inhibitory impact on the self-healing performance of asphalt [13,14]. Dong and co-workers conducted four-point bending tests and found that the self-healing performance of rock asphalt-modified asphalt is not as good as that of SBS and crumb rubber-modified asphalt [15]. Zhou and co-workers performed asphalt pull-out tests and discovered that rock asphalt-modified asphalt with appropriate proportions has a superior bonding self-healing ability [16]. Based on the literature analysis above, asphalt mixtures modified with rock asphalt have great resistance to permanent deformation. However, research on its self-healing properties remains inconclusive, and the impact of complex environmental conditions has not been adequately addressed.

As the asphalt mixture is exposed to a complex environment during service life, previous studies have only explored the impact of individual environments (temperature, ultraviolet radiation, freeze–thaw, etc.) on fatigue and self-healing performance. Zhang and co-workers undertook frequency sweep tests on aged asphalt, revealing an increase in the critical temperature of self-healing and a deterioration in self-healing ability after aging [17]. Li and co-workers investigated various wavelengths of UV aging on asphalt, uncovering a deeper aging degree under short-wave UV light irradiation, with UV light inducing changes in the chemical composition of asphalt materials [18,19,20]. Cui and co-workers examined the aging process of the asphalt FAM mixture, discovering that aging adversely impacts the self-healing ability of the asphalt FAM mixture [21]. Tarefder and co-workers observed a reduction in the creep stiffness of the asphalt FAM mixture due to freeze–thaw conditions, as determined by indirect tensile strength tests [22]. Fan and co-workers conducted indirect tensile fatigue tests, revealing a decrease in the fatigue life of specimens as the number of freeze–thaw cycles increased [23]. Zhang and co-workers, through semicircular bending tests, identified a decline in the multiple self-healing abilities of the asphalt mixture after exposure to freeze–thaw conditions [24]. Kavussi and co-workers investigated microwave-induced healing, determining that freeze–thaw has an adverse effect on microwave-induced healing [25]. It is noteworthy that all the aforementioned studies exclusively focused on individual factors, neglecting the consideration of a complex environment.

In summary, the following are some of the limitations of the earlier research:

(1) These studies exclusively concentrate on the fatigue performance of rock asphalt, resulting in a lack of research on fatigue life, considering the self-healing aspect. Furthermore, the conclusions drawn from self-healing studies are inconsistent. It is essential to conduct research on rock asphalt modified asphalt that simultaneously evaluates both fatigue and self-healing performance. In addition, the existing methods for assessing fatigue and self-healing performance basically rely on test conditions. Varied test conditions for the same material may produce different results, which will have a great impact on the consistency of test conclusions. Therefore, it is imperative to adopt an evaluation method independent of test conditions.
(2) The current research on rock asphalt is usually concentrated on asphalt binder or asphalt mixture; there is a lack of research focus on fine aggregate matrix (FAM). FAM is an important part of the asphalt mixture, and cracks generally start in the FAM system [26].
(3) These studies only focus on a single environmental factor, but the environment is more complicated during pavement service life. For example, loading from cars, short-term aging from transportation and paving, aging from UV lights, and freeze–thaw all cause damage to the road. In order to truly connect the use of modified rock asphalt materials with the actual situation, it is indispensable to study the influence of the multifaceted environment.

In summary, the primary objective of this study is to assess and compare the impact of complex environmental conditions on the fatigue and self-healing properties of FAM mixtures containing three kinds of rock asphalts (QC, UM, and Buton). In order to reach this purpose, this study focuses on the following areas: (1) the impact of rock asphalt on the fatigue and self-healing performance of FAM; (2) the impact of UV aging, freeze-thaw, and UV aging–freeze–thaw on the fatigue and self-healing performance of FAM; and (3) analysis of the internal causes of the above impacts.

## 2. Materials and Methods

### 2.1. Materials

#### 2.1.1. Asphalt Binder

In this study, six types of asphalt binder, as shown in Table 1, were selected to produce FAM mixtures. Binder A was #70 virgin asphalt; binders B to E were all virgin asphalt binder modified with rock asphalt (QC, UM, and Buton); the concentration of rock asphalt was selected based on the previous studies [2]. Binder F was a kind of high-modulus natural binder. The technical indices of the above asphalt binder tested according to JTG E20-2011 are listed in Table 2 [27].

#### 2.1.2. Aggregates

**Figure 1:**
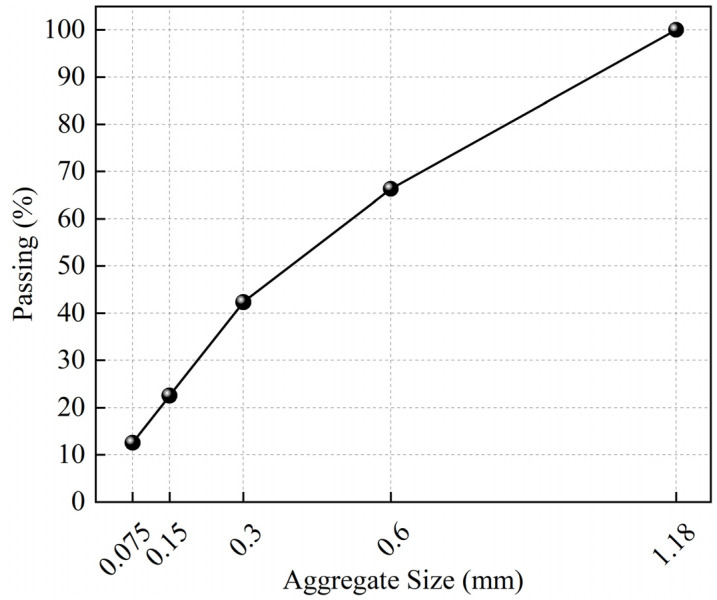
Gradation of aggregates used in the FAM mixtures.

The limestone aggregates and fillers used in this study were from Yuzhou, whose basic properties are presented in Table 3 according to JTG E42-2005 [28], and the gradation of aggregates used in the FAM mixtures is as shown in Figure 1. The detailed steps of the design methodology for rock asphalt FAM mixes can be found in Li et al. [1].

### 2.2. Sample Preparation

**Figure 2:**
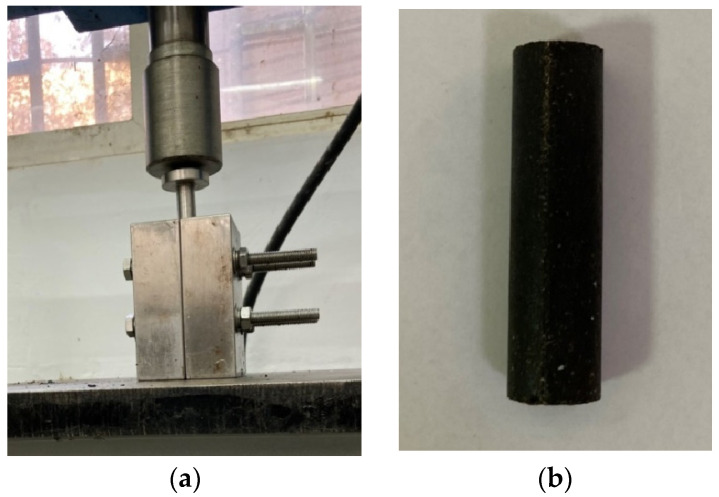
Specimen fabrication process. (**a**) Pression molding process; (**b**) cylindrical specimen.

Virgin asphalt binder was first modified with rock asphalt, then subjected to a rotational thin film oven test (RTFOT) with SH/T 0736-2003 to simulate short-term aging [29], and finally, it was mixed with aggregates in aluminum cans to produce the FAM mixture. The control mix was prepared using 89% of fine aggregates and 11% of virgin asphalt binder by total weight of the mixture, and FAM mixtures with rock asphalts were prepared by substituting an appropriate amount of fines and binders with the rock asphalt. The detailed steps of the design methodology for rock asphalt FAM mixes can be found in Li et al. [1]. The loose FAM mixture was then compacted into cylindrical specimens (12.5 mm in diameter and 50 mm in height) with an air void content close to 0 using a static press. The specimens were then cut equally at both ends to obtain cylinders with a height of 45 mm for the tests. Table 4 presents the volumetric properties of the six different short-term aged FAM mixtures prepared for this study according to JTG E20-2011 [27]. Figure 2a presents the compaction process of the cylindrical specimens, while Figure 2b presents the cylindrical specimens after cutting.

### 2.3. Laboratory Testing

#### 2.3.1. Viscoelastic Continuum Damage Theory (VECD)

The methods used to evaluate the damage and healing characteristics of asphalt materials are significantly influenced by the testing conditions, encompassing test methods, loading modes, and stress–strain levels. Additionally, prevailing methodologies commonly rely on the testing of isolated asphalt specimens, overlooking considerations of stress states within the asphalt mixture system. Consequently, relying on current evaluation methods to determine the damage and healing performance of asphalt materials cannot be considered an accurate representation of their true material properties. In response to these limitations, some researchers have suggested employing the potential theory, or the viscoelastic continuum damage theory (VECD), to investigate the fatigue resistance of asphalt materials [26]. Importantly, this theoretical framework remains independent of experimental conditions, such as static or dynamic test methods, stress or strain control loading modes, and stress–strain levels. Therefore, adopting this theory provides an effective means of mitigating the historical dependence on testing conditions observed in traditional methods.

Viscoelastic continuous damage theory (VECD) uses the functional relationship C(S) between the pseudo-stiffness *C* and the internal state variable *S* (Equation (1)) to study the cumulative fatigue damage of asphalt mixtures [30,31,32,33,34,35,36,37,38]. The internal state variable *S* represents the decrease in stiffness of the material under load; that is, *S* represents the overall damage inside the material.

(1) $$S_{N}≅S_{0}+\sum_{N=1}^{K}\left[({-\frac{I}{2}{\cdot \left({∆C}_{N}\right)\cdot \left(\varepsilon^{R}\right)}^{2})}^{\frac{\alpha }{1+\alpha }}\cdot {{∆t}_{N}}^{\frac{1}{1+\alpha }}\right]$$

where $S_{0}$ represents an internal variable representing the initial damage level of the material, which is usually assumed to be 0. I represents the initial value of the pseudo stiffness, which is introduced here to reduce the difference between samples. Normally, its value is 0.9~1.1. α represents the damage evolution rate of the material, which is related to the creep property of the asphalt mixture and is usually valued as (1 + 1/*m*) or (1/*m*), where *m* is the slope of the linear viscoelastic response equation in the logarithmic coordinate system. $C_{N}$, $\varepsilon_{N}$, and $t_{N}$ represent the pseudo stiffness, pseudo strain, and time of the *N^th^* cycle, respectively. According to the generalized elasto-viscoelastic correspondence principle (CP-II) [39] and the corresponding approximate calculation method [40], the pseudo stiffness C and pseudo strain are calculated according to Equations (2) and (3), respectively.

(2) $$\varepsilon^{R}=\varepsilon_{0}\cdot \frac{{\left|G^{*}\right|}_{LVE}}{G^{R}}$$

(3) $$C_{N}=\frac{{\left|G^{*}\right|}_{N}}{{\left|G^{*}\right|}_{LVE}}$$

where ${\left|G^{*}\right|}_{LVE}$ represents the dynamic shear modulus of viscoelastic material in the linear viscoelastic range; $G^{R}$ is the reference modulus, and you can choose any value; $C_{N}$ represents the pseudo stiffness *C* of the *N^th^* load cycle; and ${\left|G^{*}\right|}_{N}$ is the *N^th^* load cycle dynamic shear modulus.

Relevant scholars have further proved that the C(S) function represents the unique properties of the material and has nothing to do with test conditions such as test method (static or dynamic), loading mode (stress control or strain control), or stress–strain level [41]. Therefore, this theory can effectively eliminate the dependence of traditional methods on experimental conditions. Currently, asphalt binder, FAM mixture, and asphalt mixture fatigue performance evaluation and prediction have been successfully accomplished with VECD [2].

#### 2.3.2. Fatigue Damage and Self-Healing Tests

**Figure 3:**
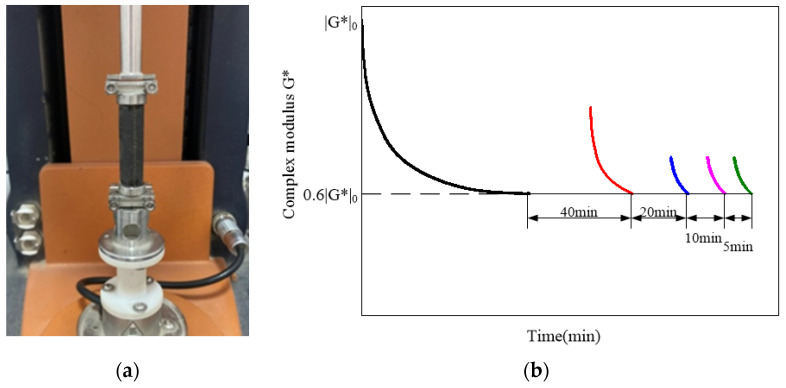
Installation and loading of specimens. (**a**) DSR with a cylindrical FAM specimen; (**b**) schematic illustration of fatigue-healing tests.

The creep test on FAM specimens was first carried out with a stress amplitude of 10 kPa for 20 s at 25 °C using the Dynamic Shear Rheometer (DSR). After the creep test, the specimen was allowed to recover for 20 min without an external load being applied. Subsequently, the specimen underwent a 90-second time-sweep test at 25 °C with a strain amplitude of 0.006% and a frequency of 5 Hz. After the linear viscoelasticity tests above were completed, the specimen was allowed to heal for 20 min under 25 °C conditions. Then, the specimen was subjected to fatigue-healing tests in stress-controlled mode. First, the specimen was sheared at a frequency of 5 Hz until the dynamic shear modulus |G*| decreased to 60%|G*|_0_. At this moment, a 40-minute rest break was established. Following the rest period, loading was continued until the specimen’s modulus decayed to 60%|G*|_0_, and another 20-minute rest period was introduced thereafter. By analogy, the process was repeated, except that the subsequent rest periods are 10 min and 5 min in length, respectively. The details of this procedure can be found in Karki et al. [42] and Li et al. [2]. The DSR with the cylindrical FAM specimen is as shown in Figure 3a, and the detailed test schematic is as shown in Figure 3b.

The C-S curve includes important information about fatigue damage characteristics and can be used to compare the fatigue cracking resistance of two different materials through appropriate application. The detailed information can be found in Li et al. [2]. Therefore, a fatigue life prediction model (without and with rest periods) based on VECD theory (as shown in Equations (4), (5), (6), and (7), respectively) was employed in the study.

(4) $$N_{u}≅f\cdot \sum_{k=1}^{C_{u}}\left[{\left\{-\frac{1}{2}\cdot \left(\Delta C_{u,k}^{R}\right){\left(\varepsilon^{R}\right)}^{2}\right\}}^{-\alpha }\cdot {\left(\Delta S_{u,k}\right)}^{1+\alpha }\right]$$

where $N_{u}$ represents the number of load cycles without rest period; f represents input loading frequency; $\Delta C_{u,k}^{R}$ represents the pseudo-stiffness at the *k^th^* loading cycle; $\varepsilon^{R}$ represents pseudo-strain; and α indicates the rate of damage evolution of the material.

(5) $$S_{u,A}≅\left(1-HI\right)\cdot S_{u,B}$$

(6) $$\Delta N_{BA}=N_{B}-N_{A}$$

(7) $$N_{F}=N_{B}+\Delta N_{BA}$$

In Equations (5)–(7), $S_{u,A}$ indicates the remaining damage parameter after the rest period; $S_{u,B}$ indicates a damage parameter of 60% pseudo stiffness after first loading; N_A_ indicates loading cycles corresponding to the remaining damage after the rest period; N_B_ indicates loading cycles after the first loading to 60% damage; $\Delta N_{BA}$ indicates the difference in the cycles required for the pseudo-stiffness to return to the pseudo-stiffness level before the rest period; and $N_{F}$ indicates the fatigue life of the FAM mixture considering the effect of self-healing.

**Figure 4:**
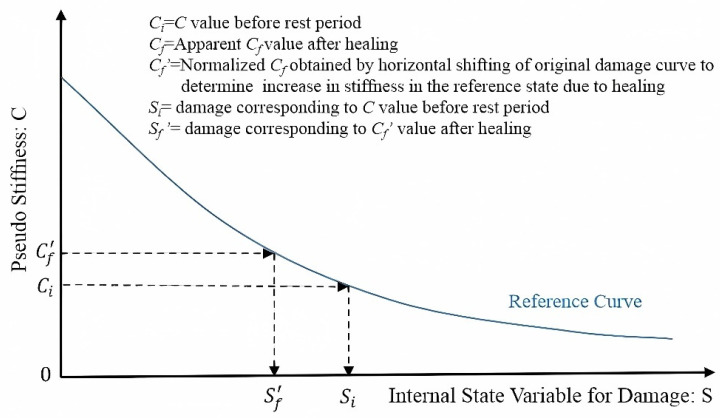
Schematic diagram of self-healing index HI calculations [42].

The detailed analytical procedure to determine the self-healing properties of FAM mixtures can be found in Karki et al. [42] and Li et al. [2]. The healing index is quantified as follows:

(8) $$\mathrm{HI}=\frac{S_{i}-{S_{f}}^{\prime }}{S_{i}}\times 100\%$$

where HI represents healing index, %; *S_i_* represents the cumulative damage parameter prior to the rest period; and *S_f_′* represents the damage parameter corresponding to the normalized pseudo stiffness *C_f_*′ after self-healing. Figure 4 illustrates the calculation of the damage parameter *S* using the reference C(S) curve.

#### 2.3.3. UV Aging Tests

Currently, there is no unified understanding or consensus on the ultraviolet (UV) light aging of asphalt binder. This study conducted UV tests based on the previous research findings [43,44]. It is worth mentioning that, before conducting the UV tests, RTFOT was carried out on the asphalt samples. Then, the aged binders were put into the UV aging oven at 35 °C for 72 h, 144 h, and 216 h, respectively. The UV light is 30 cm away from the specimens, the UV light irradiation intensity is 120 W/m^2^, and the asphalt samples were placed on a sample holder with a diameter of 500 mm and a rotational speed of 5 r/min.

#### 2.3.4. Freeze-Thaw Tests

In this study, the FAM specimens were employed for freeze–thaw tests. The freeze–thaw cycle procedure was according to JTG F40-2004 [45], and three steps were concluded for each freeze–thaw test cycle: vacuum water absorption, freezing for 2 h at −18 ± 2 °C, and thawing for 1 h at 60 ± 0.5 °C. In the first phase, the FAM specimens were put into water at a temperature of 25 °C and a pressure of 98 kPa for 15 min. In the second phase, the specimens were tightly wrapped in plastic wrap and then put into the low-temperature cabinet for 2 h. In the third phase, the specimens were put into the constant-temperature water bath for 1 h. The test was designed with three freeze–thaw cycles (25 cycles, 50 cycles, and 75 cycles) to evaluate the impact of different cycle numbers on fatigue and self-healing capabilities.

#### 2.3.5. UV Aging and Freeze–Thaw Coupling Tests

In order to simulate the actual conditions during the pavement’s service life, UV aging and freeze–thaw coupling tests were conducted. Firstly, the asphalt binders were UV-aged using the method in Section 2.3.3, and then FAM specimens were produced by the UV-aged binders, and finally, the UV-aged FAM specimens were subjected to the freeze–thaw test. The following two different groups were employed in this study: UV aging for 72 h and freeze–thaw for 25 cycles, and UV aging for 216 h and freeze–thaw for 25 cycles.

#### 2.3.6. Microstructure Tests

In this study, atomic force microscope (AFM) tests and Fourier transform infrared spectroscopy (FTIR) tests were conducted to analyze the microstructure and functional group changes of asphalt binders. The AFM specimens were prepared on glass slides and tested by Dimension Fastscan 03040155 from Bruker, Berlin, Germany in tapping mode [43]. The FTIR specimens were tested at wavenumbers ranging from 4000 cm^−1^ to 400 cm^−1^ [46].

In order to analyze the changes in functional groups after UV aging, the contents of carbonyl and sulfoxide groups were used as factors. According to French testing method ME 69 of LPC [47], the content of -CH- functional groups is used as the base value, and the ratio of carbonyl and sulfoxide groups to it is used as a measure of the degree of aging. The calculation method is shown in Equations (9) and (10).

(9) $$I_{C=O}=\frac{A_{C=0}}{A_{C-H}}$$

(10) $$I_{S=O}=\frac{A_{S=0}}{A_{C-H}}$$

where $A_{C=0}$ indicates the peak area of carbonyl (C=O) absorption at 1700 cm^−1^; $A_{S=0}$ indicates the peak area of sulfoxide (S=O) absorption at 1031 cm^−1^; and $A_{C-H}$ indicates the peak areas of saturated -C-H- absorption at 1461 cm^−1^ and 1376 cm^−1^.

## 3. Results and Discussion

### 3.1. Results of Fatigue Damage and Self-Healing Healing Tests

**Figure 5:**
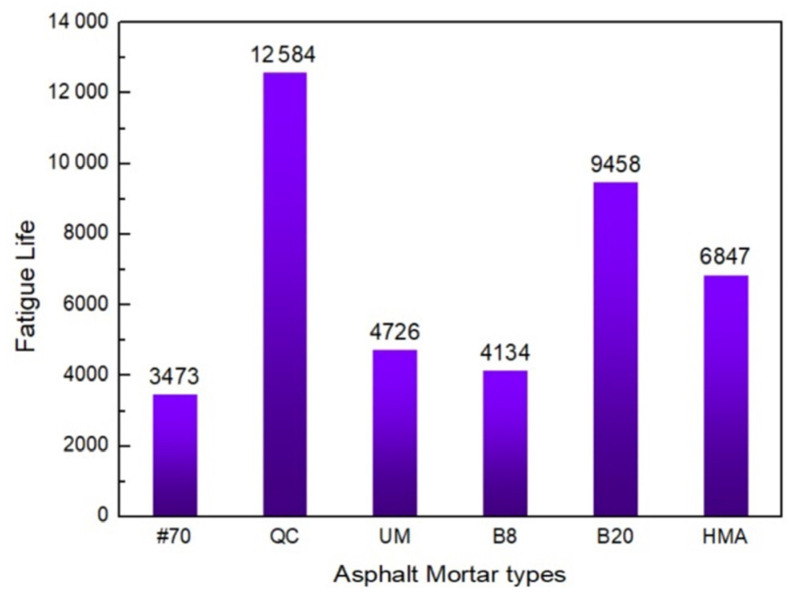
Fatigue life prediction results of FAM mixtures.

In this study, Equation (5) and a stiffness threshold for failure (e.g., a 60% reduction in C-value) were used to forecast the fatigue life of the FAM mixtures and to evaluate the relative fatigue performance of the various composites. The fatigue lives of several FAM mixtures are displayed in Figure 5.

By comparing the data in Figure 5, it can be seen that the fatigue life of QC, UM, 8%, and 20% Buton rock asphalt FAM mixtures increased by 265%, 37%, 20%, and 175%, respectively, compared to the #70 asphalt FAM mixture. The longer fatigue life exhibited by rock asphalt FAM mixtures is attributed to their higher stiffness, which is beneficial for resisting deformation failure, which is consistent with previous research findings [1]. In addition, the fatigue life of the 20% Buton rock asphalt FAM mixture increased by 129% compared with the 8% Buton rock asphalt FAM mixture.

**Figure 6:**
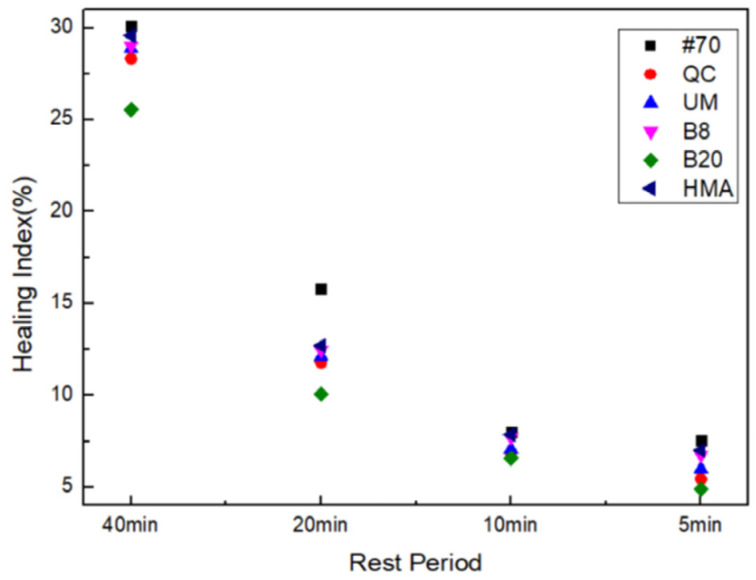
Healing index of FAM mixtures.

The self-healing properties of FAM mixtures were represented by the healing index (HI) calculated by Equation (8). Figure 6 shows the HI calculation results.

Figure 6 shows that the healing index of rock asphalt FAM mixtures was lower than that of the #70 FAM mixture. This phenomenon can be attributed to the fact that rock asphalt contains a higher proportion of asphaltenes compared to virgin asphalt, which hinders the flowability of rock asphalt, resulting in lower self-healing abilities of rock asphalt FAM mixtures, which is consistent with previous research findings [48]. At the same concentration of 8%, the healing index of the QC rock asphalt FAM mixture was the lowest. It can also be seen that increasing the concentration of rock asphalt has a negative effect on the self-healing performance of FAM mixtures. In addition, the self-healing performance of the HMB FAM mixture was worse than that of the #70 FAM mixture but better than that of other types of rock asphalt FAM mixtures.

**Figure 7:**
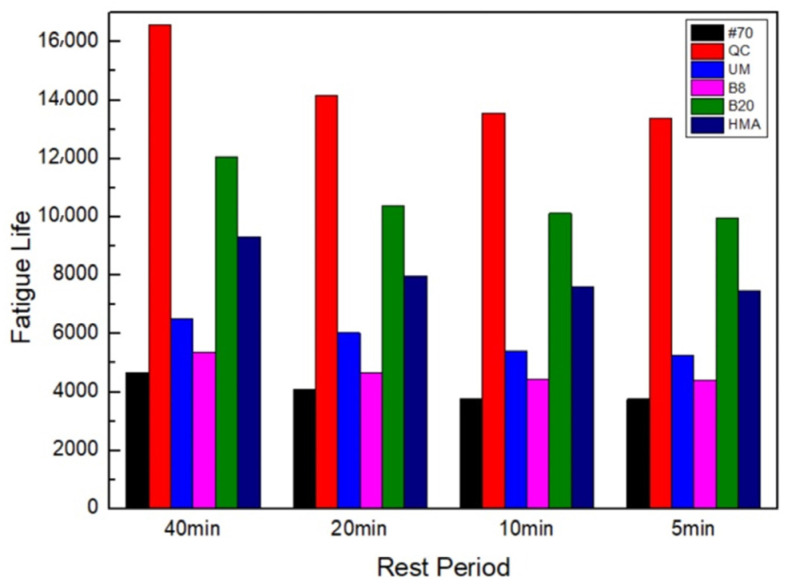
Fatigue life prediction results considering self-healing.

This study has examined the fatigue life of asphalt FAM mixes that take self-healing into consideration in order to provide a basis for the real service life of the pavement. The fatigue life of asphalt FAM mixes that included self-healing was calculated using Equations (5)–(7). Figure 7 shows the results.

Figure 7 shows that the order of fatigue life of asphalt FAM mixtures had not changed after considering the influence of self-healing, indicating that although rock asphalt had a negative impact on the self-healing performance, the fatigue life considering self-healing of rock asphalt FAM mixtures was still higher than that of the #70 FAM mixture. Moreover, the QC rock asphalt FAM mixture still exhibited the longest fatigue life. Therefore, rock asphalt can be used as a more practical modifier in actual use to improve the service life of asphalt pavement.

### 3.2. Results of Fatigue and Self-Healing Tests after UV Aging

**Figure 8:**
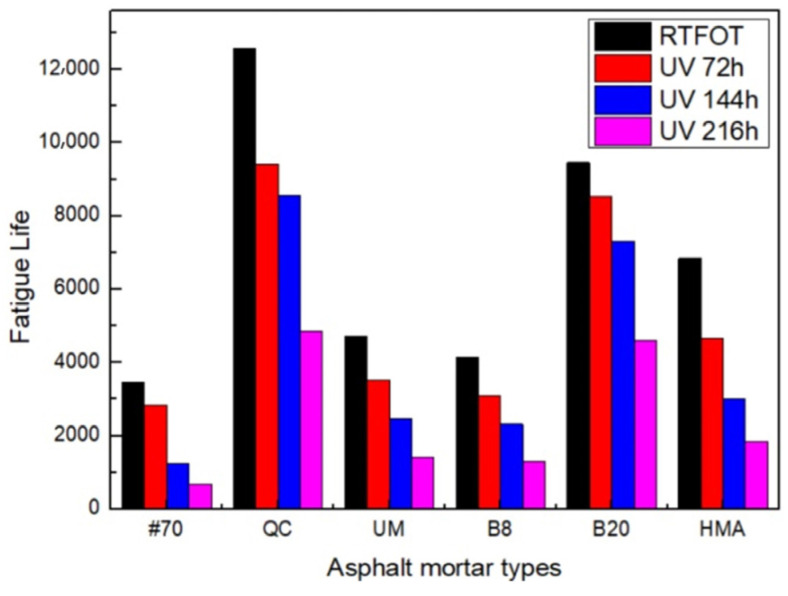
Fatigue life prediction results after UV aging.

The results of fatigue life after UV aging are shown in Figure 8.

Figure 8 shows that the fatigue life of each asphalt FAM mixture is reduced after UV aging. The fatigue life of the #70 asphalt FAM mixture decreased most rapidly, with the fatigue life after 216 h of UV aging being only 20% of that after RTFOT. This is attributed to the sensitivity of aromatics and resins in asphalt to ultraviolet light, which easily undergo photo-oxidation reactions to form asphaltene, leading to the degradation of asphalt performance [49]. The increase in the effect of UV aging on the fatigue life of the asphalt FAM mixture was not simply linear. For example, the fatigue life of the 8% QC rock asphalt FAM mixture UV-aged for 144 h was 9.3% lower than that for 72 h, while the fatigue life of that UV-aged for 216 h was 43% lower than that for 144 h. At the same concentration of 8%, the fatigue life of the Buton rock asphalt FAM mixture decreased significantly. Compared to the fatigue lives of different concentrations of UV-aged Buton rock asphalt FAM mixtures, the effect of UV aging on the 20% Buton rock asphalt FAM mixture was smaller. Thus, it is shown that the fatigue life of rock asphalt FAM mixes became less affected by UV aging as the rock asphalt concentration increased. UV aging had the greatest impact on the fatigue performance of the HMA FAM mixture compared with other varieties of rock asphalt. From the above results, it can be observed that different types of asphalt exhibit variations in resistance to UV aging, which is attributed to differences in the content of aromatic compounds and resins in the asphalt [50].

**Figure 9:**
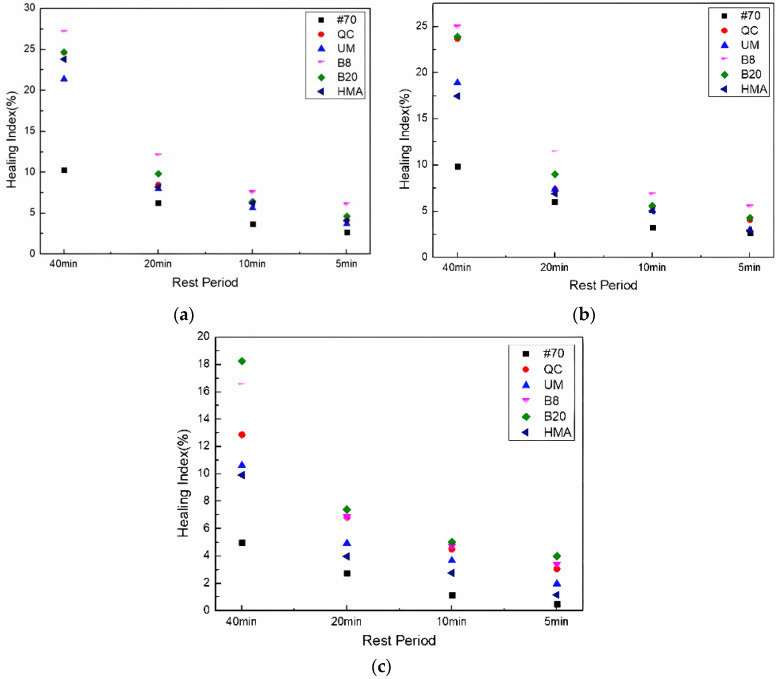
Healing index after UV aging. (**a**) UV aging for 72 h; (**b**) UV aging for 144 h; and (**c**) UV aging for 216 h.

The HI calculation results of asphalt FAM mixtures after UV aging are shown in Figure 9.

Figure 9 shows that after UV aging, the healing index of different asphalt FAM mixtures decreased, and the order of the healing index under different rest periods was the same. As the UV aging hours increased, the healing index of the FAM mixtures decreased. For example, after UV aging for 216 h, the healing index of the #70 asphalt FAM mixture was the lowest. The decrease was the most obvious when it increased from 144 h to 216 h. It can also be seen that, similar to the effect of UV aging on fatigue life, the Buton rock asphalt FAM mixture was most affected by UV aging among the same concentration of rock asphalt FAM mixtures. The healing indexes of the 8% Buton rock asphalt FAM mixture UV-aged for 72 h and 144 h were higher than those of the 20% Buton rock asphalt FAM mixture. With the increase in UV aging time, after 216 h of UV aging, the healing index of the 20% Buton rock asphalt FAM mixture was higher than that of the 8% Buton rock asphalt FAM mixture.

**Figure 10:**
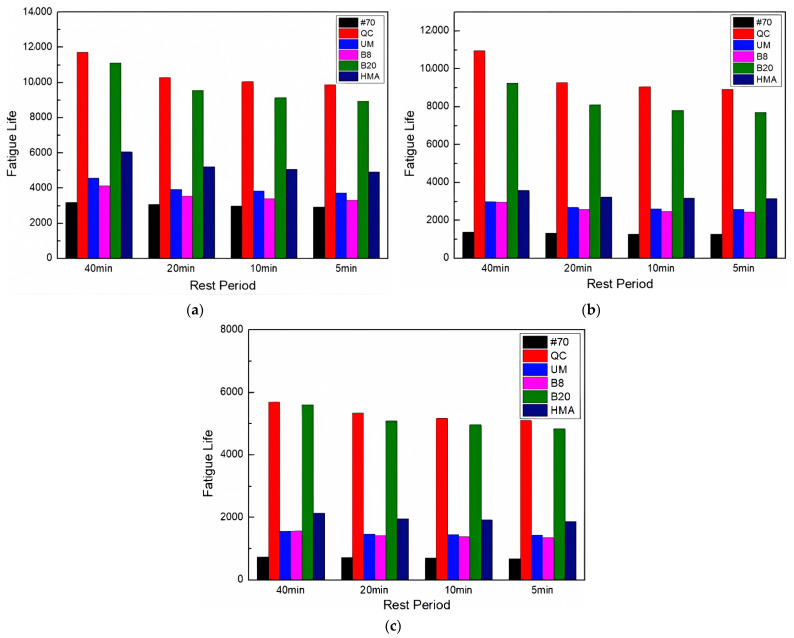
Fatigue life prediction considering self-healing after UV aging. (**a**) UV aging for 72 h; (**b**) UV aging for 144 h; and (**c**) UV aging for 216 h.

The fatigue life prediction considering self-healing is shown in Figure 10.

Figure 10 compares the fatigue life of various asphalt FAM mixes under the influence of UV aging; although the overall trend remained the same, their gap changed. The gap between the fatigue life of the 20% Buton rock asphalt FAM mixture and that of the QC rock asphalt FAM mixture has narrowed. The gap between the fatigue life of the 8% Buton rock asphalt FAM mixture and that of the UM rock asphalt FAM mixture has also narrowed. Moreover, the fatigue life of the 20% Buton rock asphalt FAM mixture was much higher than that of the 8% Buton rock asphalt FAM mixture. It can be concluded that UV aging changed the fatigue life gap between asphalt FAM mixtures, and under UV aging conditions, the ability of asphalt FAM mixtures to resist UV aging can be enhanced by increasing the concentration of rock asphalt.

### 3.3. Results of Fatigue and Self-Healing Tests after Freeze–Thaw

**Figure 11:**
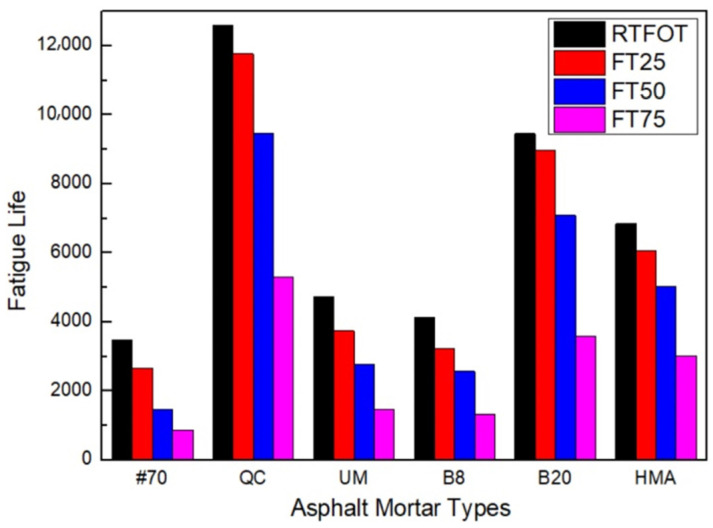
Fatigue life prediction results after freeze–thaw cycles.

The fatigue life prediction result of asphalt FAM mixtures after freeze-thaw is shown in Figure 11. FT25, FT50, and FT75 represent 25, 50, and 75 freeze–thaw cycles, respectively.

Figure 11 shows that the fatigue life of all asphalt FAM mixtures decreased after freeze–thaw. This is attributed to the expansion stress, internal stress, and temperature stress generated by the expansion and melting of ice, which disrupt the structure of the FAM mixture [51]. The fatigue life of the #70 asphalt FAM mixture decreased mostly by 76% after 75 freeze–thaw cycles, significantly higher than that of the rock asphalt FAM mixtures. With an identical quantity of freeze–thaw cycles, the fatigue life of the UM rock asphalt FAM mixture decreased the fastest, while the fatigue life of the QC rock asphalt FAM mixture decreased the slowest. The fatigue life of 8% and 20% Buton rock asphalt FAM mixtures was reduced by 22% and 5% when the freeze–thaw cycles increased from 0 to 25. This indicates that freezing and thawing had the greatest effect on the fatigue properties of the #70 asphalt FAM mixture, and the effect on the rock asphalt FAM mixture was ranked as follows: UM > Buton > QC. For the same species of rock asphalt, the impact of freezing and thawing on the fatigue properties of the rock asphalt FAM mixture decreased as the doping level increased. After 25 freeze–thaw cycles, the fatigue life of the HMA FAM mixture was reduced by 11%. Its effect on the fatigue performance of the HMA FMA mixture was in the middle between the UM rock asphalt FAM mixture and the QC rock asphalt FAM mixture as compared to other varieties of rock asphalt FAM mixture.

**Figure 12:**
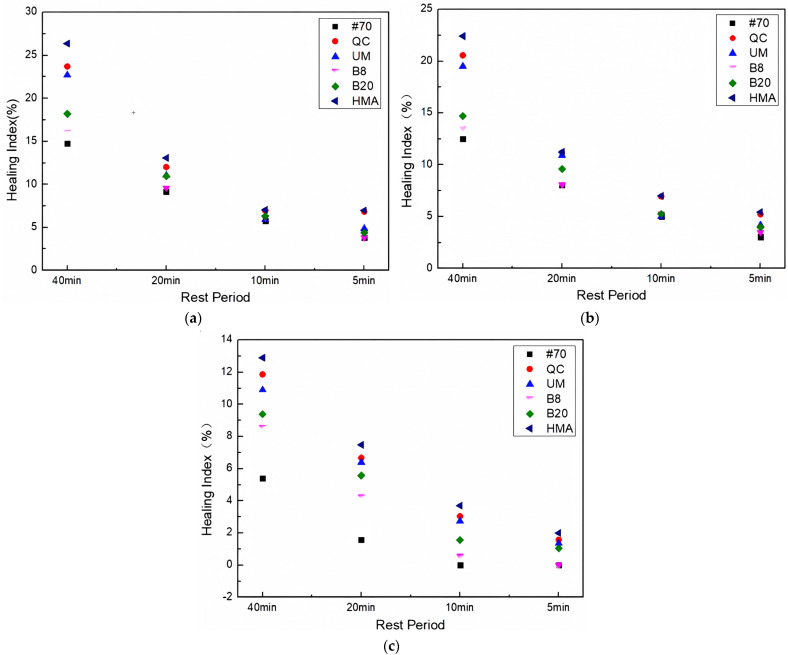
Healing index after (**a**) FT25, (**b**) FT50, and (**c**) FT75.

The healing index of asphalt FAM mixtures after freeze–thaw tests is shown in Figure 12.

Figure 12 shows that the healing index of all asphalt FAM mixtures decreased after freeze–thaw cycles. Compared with the change in the healing index of asphalt FAM mixtures between 0 and 25 and 25 and 50 freeze–thaw cycles, the decrease in the healing index of asphalt FAM mixtures was more obvious when the number of freeze–thaw cycles increased from 50 to 75. The healing index of the #70 asphalt FAM mixture decreased more obviously after freeze–thaw, while among the three rock asphalt FAM mixtures with the same concentration, the healing index of the Buton rock asphalt FAM mixture had the largest change, and the healing index of the QC rock asphalt FAM mixture had the smallest change. In addition, comparing different concentrations of Buton rock asphalt FAM mixtures, it was discovered that the influence of freeze–thaw on the self-healing ability of asphalt FAM mixtures decreased for the same rock asphalt as the concentrations of rock asphalt blended increased. While among rock asphalt FAM mixtures, the HMA FAM mixture had the highest healing index under the same freeze–thaw cycles. This indicates that freezing and thawing had the least effect on the self-healing ability of the HMA FAM mixes compared to the other varieties of rock asphalt FAM mixtures.

**Figure 13:**
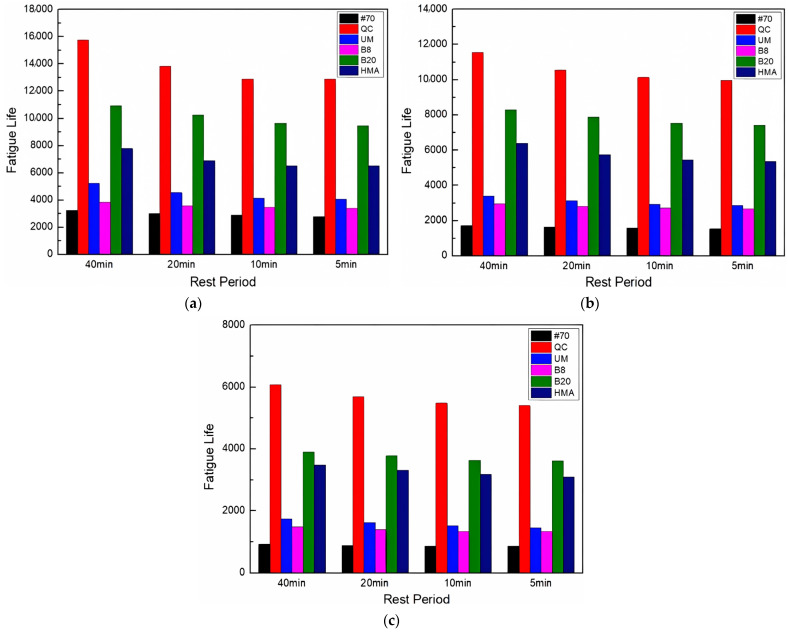
Fatigue life prediction considering self-healing after (**a**) FT25, (**b**) FT50, and (**c**) FT75.

The fatigue life considering the self-healing of asphalt FAM mixtures after freeze–thaw cycles is shown in Figure 13.

Figure 13 shows that after considering the effect of self-healing, the order of fatigue lives did not change. However, due to the different sensitivity of the self-healing ability to freeze–thaw cycles, there were gaps in the life of the FAM mixtures. The fatigue life of the #70 asphalt FAM mixture, considering self-healing, and that of several rock asphalt FAM mixtures had an increased gap. This is because the fatigue life and healing index of the #70 asphalt FAM mixture were affected by freeze–thaw, which was larger than that of several rock asphalt FAM mixtures. Therefore, the following conclusion can be drawn: Considering the influence of freeze–thaw, the disparity in fatigue life among FAM mixtures was altered, with rock asphalt demonstrating the capacity to mitigate the impact of freeze–thaw cycles on the fatigue life of these FAM mixtures. By comparing the fatigue lives of 8% and 20% Buton rock asphalt FAM mixtures, it can be seen that the higher concentration of rock asphalt FAM mixture was more resistant to freezing and thawing.

### 3.4. Results of Fatigue and Self-Healing Tests after UV Aging–Freeze–Thaw

**Figure 14:**
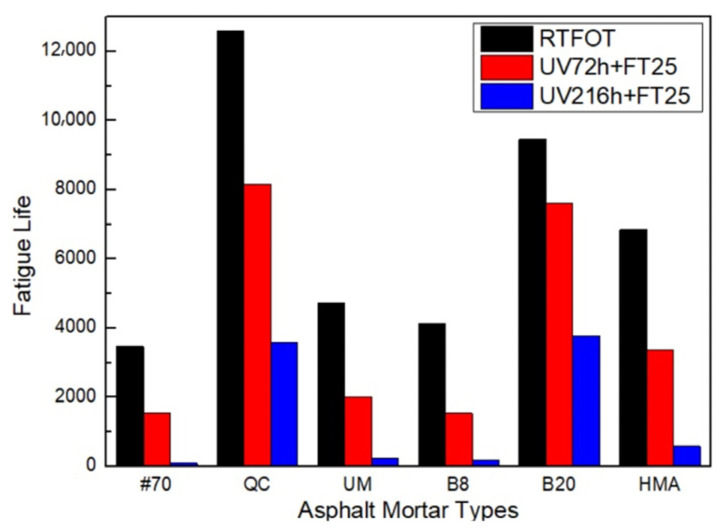
Fatigue life prediction results after the UV aging–freeze–thaw process.

The calculation results of fatigue life after UV aging–freeze–thaw process are shown in Figure 14.

Figure 14 shows that under the UV aging–freeze–thaw process, the fatigue life of the #70 asphalt FAM mixture decreased mostly by 98% after UV216 h + FT25, while the 20% Buton rock asphalt FAM mixture decreased least by 61%. While among the three types of rock asphalt FAM mixtures with the same concentration, the fatigue life of the Buton rock asphalt FAM mixture was most obviously affected by UV aging–freeze–thaw, while the QC rock asphalt FAM mixture was least affected. In addition, from RTFOT to UV72 h + FT25, the fatigue life of the 8% Buton rock asphalt FAM mixture was reduced by 53%, and the fatigue life of the 20% Buton rock asphalt FAM mixture was reduced by 19%. The fatigue life reduction of the HMA FAM mixture was longer than that of the QC rock asphalt FAM mixture and lower than that of the UM rock asphalt FAM mixture. Through comparison and simple calculation, it was shown that the fatigue properties of the asphalt FAM mixture after UV aging–freeze–thaw were reduced more than those under a single factor test, and the effect of UV aging–freeze–thaw on fatigue properties was greater than the superposition of the two single factor tests.

**Figure 15:**
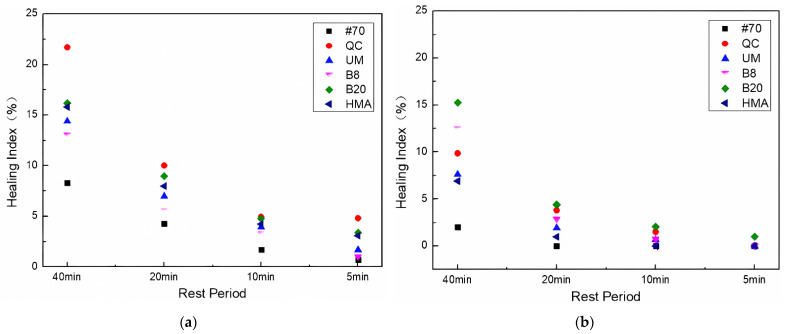
Healing index after (**a**) UV72 h + FT25 and (**b**) UV216 h + FT25.

The results of HI after the UV aging–freeze–thaw process are shown in Figure 15.

Figure 15 shows that the healing index of the #70 asphalt FAM mixture decreased the most after UV aging–freeze–thaw, and adding rock asphalt can increase the resistance of the asphalt FAM mixture to UV aging–freeze–thaw effect to a certain extent. Among the three 8% rock asphalt FAM mixtures, the QC rock asphalt FAM mixture had the largest decrease in healing index, followed by UM and Buton. Moreover, after UV aging-freeze–thaw, the healing index of 20% Buton rock asphalt FAM mixture changed from less than 8% Buton rock asphalt FAM mixture to more than 8% Buton rock asphalt FAM mixture. While from RTFOT to UV72 h + FT25, the healing index of the HMA FAM mixture reduced mostly by 51%.

**Figure 16:**
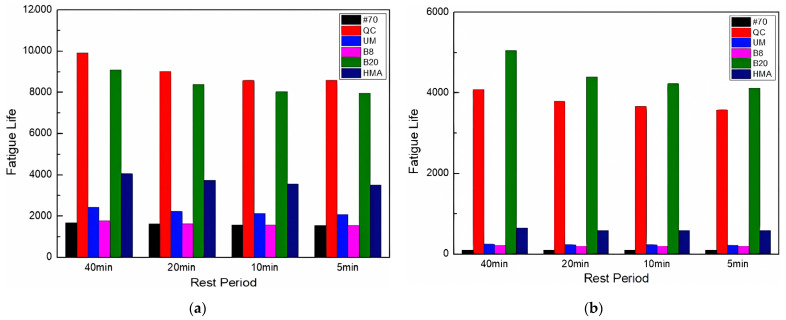
Fatigue life prediction considering self-healing after (**a**) UV72 h + FT25 and (**b**) UV216 h + FT25.

The fatigue life considering self-healing after the UV aging–freeze–thaw process is shown in Figure 16.

As is shown in Figure 16, since the fatigue life and healing index of the 20% Buton rock asphalt FAM mixture were less affected by UV aging–freeze–thaw, the gap between the fatigue life considering self-healing of the 20% Buton rock asphalt FAM mixture and the QC rock asphalt FAM mixture was narrowing. The fatigue life of the 20% Buton rock asphalt FAM mixture was lower than that of the QC rock asphalt FAM mixture after UV72 h + FT25 but became longer than that of the QC rock asphalt FAM mixture after UV216 h + FT25.

### 3.5. Results of Microstructure Tests

#### 3.5.1. AFM Tests

**Figure 17:**
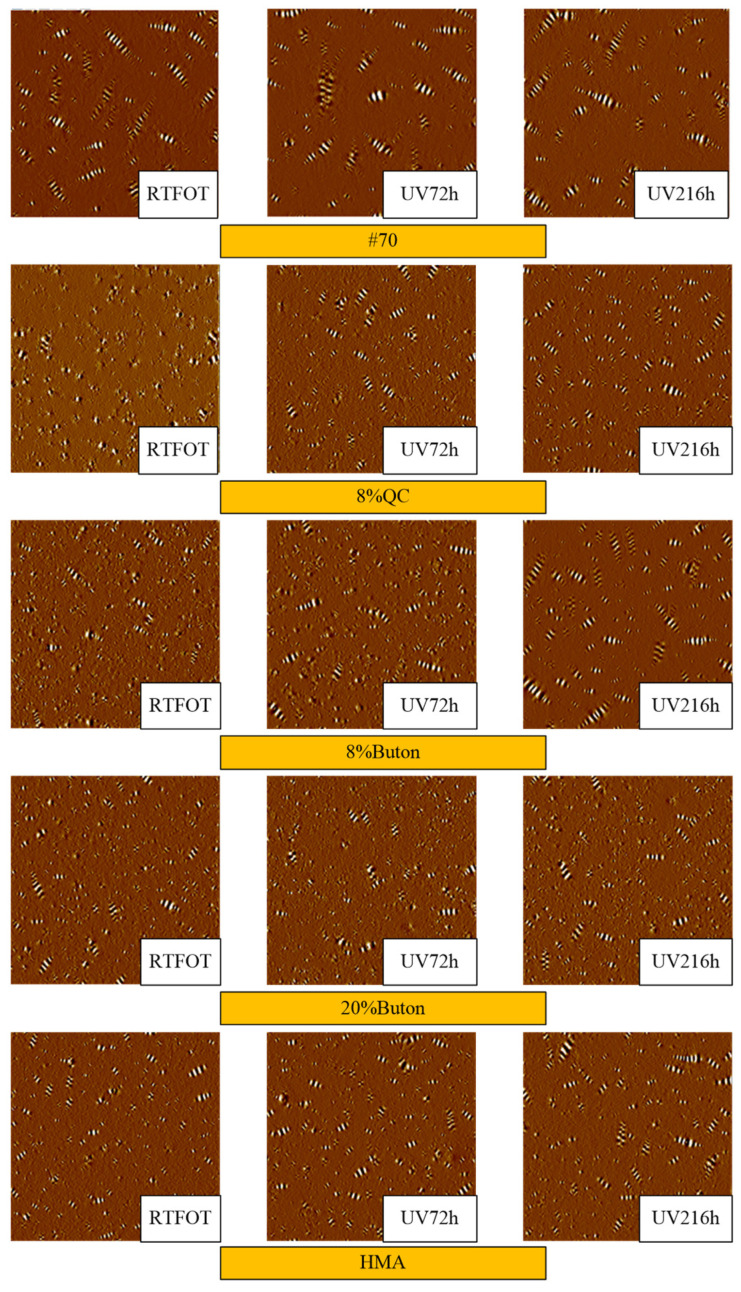
AFM images.

AFM results are shown in Figure 17.

AFM images are composed of three phases, which include the bee-structure, dispersed domain, and flat matrix. It is generally believed that the bee-structure is mainly composed of wax crystals. The asphaltene has a strong polarity; under the attraction of the strong polarity, the wax component and the asphaltene aggregate crystallize to form a bee-structure [46,52,53].

Figure 17 shows that with the increase in UV aging hours, the bee-structure all decreased, the area of the bee-structure all increased, and the distribution of the bee-structure was uneven, which caused the asphalt binder to appear stress-concentrated under load and was more likely to be destroyed under stress. Therefore, as the aging hours increased, the fatigue performance of the asphalt FAM mixture decreased significantly.

Rock asphalt-modified asphalt binders had more bee-structures but a smaller area and a more uniform distribution. The content of asphaltene and resin increased, and the content of saturate and aromatic decreased, which can cause the microscopic appearance to become rough. The rock-added asphalt became viscous, and the high molecular weight components increased, which is consistent with previous research findings [54]. As the asphaltene content increased, the number of bee-structures also increased, but the bee-structures were more evenly distributed, which increased the homogeneity of the asphalt. Therefore, the use of rock asphalt increased the asphalt’s fatigue life.

The #70 asphalt’s bee-structure had a more noticeable alteration following the same time of UV aging as the modified rock asphalt asphalts. Therefore, the fatigue performance of the rock asphalt FAM mixture after UV aging was weaker than that of the virgin asphalt FAM mixture. Similarly, the bee-structure of 20% Buton rock asphalt modified asphalt changed the smallest, which was affected the least during UV aging, so its fatigue performance was also the least affected.

#### 3.5.2. FTIR Tests

**Figure 18:**
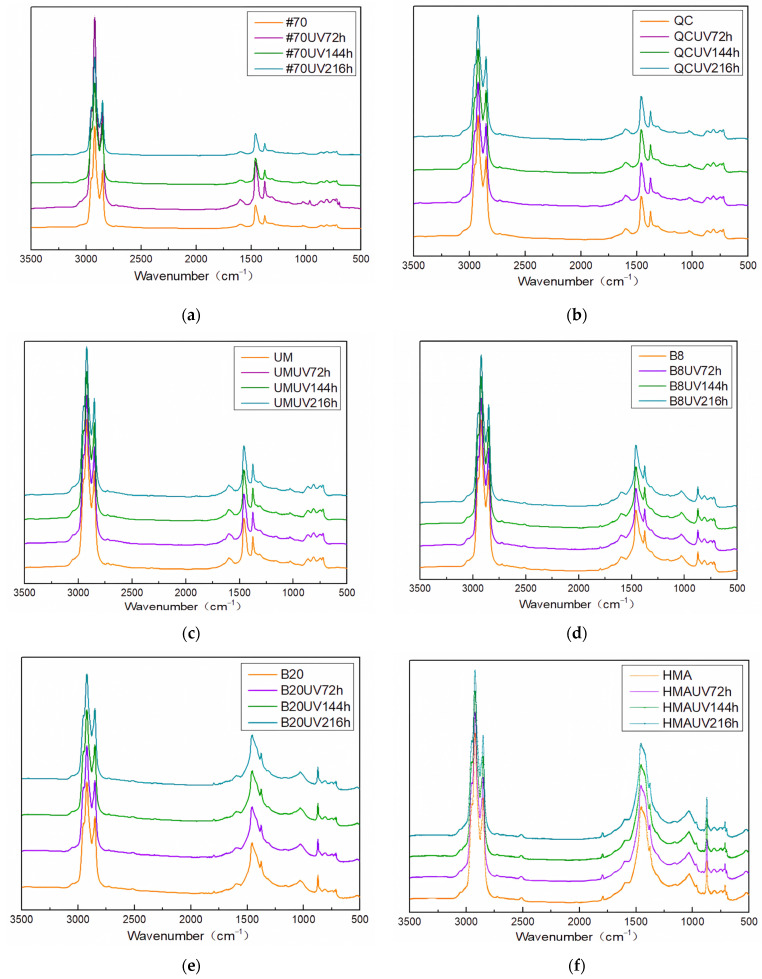
FTIR results. (**a**) #70; (**b**) QC; (**c**) UM; (**d**) B8; (**e**) B20; and (**f**) HMA.

The FTIR results are shown in Figure 18.

The absorption peaks of virgin asphalt, QC rock asphalt modified asphalt, and UM rock asphalt modified asphalt were the same, so there is only the FTIR result of virgin asphalt. Figure 18 demonstrates that the absorption peaks of modified rock asphalts were basically the same as those of virgin asphalt, indicating that the primary physical modification caused by rock asphalt on virgin asphalt. The unique absorption peaks of 8% Buton rock asphalt modified asphalt, 20% Buton rock asphalt modified asphalt, and HMA were the D_2_O stretching vibration absorption peak at 2512 cm^−1^ and the methacrylic anhydride C=O stretching vibration absorption peak at 1797 cm^−1^, which proved that these three kinds of rock asphalt modified asphalt contained more unsaturated carbon chains and amino groups and contained a certain amount of salt minerals.

**Figure 19:**
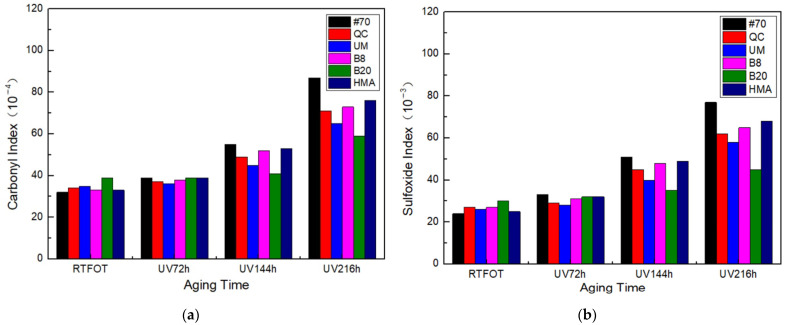
The carbonyl index and sulfoxide index. (**a**) Carbonyl index and (**b**) sulfoxide index.

The calculation results of the carbonyl index and sulfoxide index are shown in Figure 19.

Figure 19 shows that the carbonyl and sulfoxide index of rock asphalt modified asphalt was higher than that of #70 asphalt after RTFOT. This is because rock asphalt is natural asphalt, which has undergone a long period of aging in the natural environment and has a high content of carbonyl and sulfoxide groups. After UV aging, the carbonyl and sulfoxide indexes in asphalt binders increased. After 216 h of UV aging, the carbonyl and sulfoxide index of #70 asphalt increased the most, by 171% and 220%, respectively. As the UV aging hours increased, the index also gradually increased. It can be seen that as the UV aging hours increased, the degree of aging increased. In addition, the carbonyl index and sulfoxide index of the rock asphalt modified asphalt were lower than those of the #70 asphalt after UV aging, which proved that under the same UV aging hours, the aging degree of the #70 asphalt was higher than that of the rock asphalt modified asphalt. Therefore, the fatigue life and healing index of rock asphalt FAM mixtures were less affected than that of #70 asphalt FAM mixtures, corresponding to the fatigue and self-healing performance. The 20% Buton rock asphalt modified asphalt had the lowest carbonyl and sulfoxide index after UV aging by 52% and 50%, respectively, so the fatigue life and healing index of the 20% Buton asphalt FAM mixture were affected the least by UV aging.

## 4. Conclusions

In this study, through time sweep tests and data modeling based on the VECD theory, the effects of UV aging, freeze–thaw, and UV aging–freeze–thaw on the fatigue and self-healing properties of FAM mixtures containing rock asphalts were investigated. The following conclusions were drawn from this study:

(1) Rock asphalt has the ability to extend the fatigue life of FAM mixtures. Specifically, the fatigue life of the QC rock asphalt FAM mixture is 3.65 times longer than that of the 70# FAM mixture. However, these rock asphalt FAM mixtures simultaneously demonstrate weaker self-healing capabilities. As the concentration of rock asphalt increased, the fatigue life of FAM mixtures increased while their self-healing performance decreased. In consideration of self-healing properties, the rock asphalt FAM mixture still demonstrated a relatively higher fatigue life.
(2) UV aging reduced the fatigue and self-healing performance of FAM mixtures. Notably, the fatigue life of the #70 asphalt FAM mixture decreased to only 20% of its initial value after 216 h of UV exposure. As the number of UV aging hours increased, this effect also increased. Rock asphalt can resist this effect to a certain extent, and the higher the concentration, the better the resistance. Among several rock asphalts with the same concentration, the one with the strongest resistance to the effect of UV aging was UM rock asphalt.
(3) Freeze–thaw led to a reduction in both the fatigue and self-healing performance of the FAM mixture. In particular, the fatigue life of the #70 FAM mixture was decreased by 76% after 75 freeze–thaw cycles. An increase in the number of freeze–thaw cycles corresponded with a more pronounced decline in performance. Rock asphalt was found to possess an inherent capacity to mitigate the deleterious effects of freeze–thaw, with its efficacy being positively correlated with higher concentrations. Among composites with equivalent concentrations of rock asphalt, the QC rock asphalt FAM mixture exhibited the most robust resistance to freeze–thaw conditions.
(4) The fatigue and self-healing performance of the FAM mixture were reduced after UV aging-freeze–thaw. The incorporation of rock asphalt endowed the FAM mixture with an enhanced capacity to counteract the detrimental effects of UV aging–freeze–thaw, with higher concentrations of rock asphalt yielding superior outcomes. Moreover, the fatigue life of the 20% Buton rock asphalt FAM mixture was the least affected, decreasing by 61% after UV216 h + FT25. In addition, the reduction in fatigue life was more pronounced than the cumulative effects of the individual condition tests.
(5) AFM results indicate that UV aging resulted in a decrease in the number of bee-structures and an enlargement of their respective areas, whereas the incorporation of rock asphalt improved the uniformity of the bee-structure distribution, thereby improving the fatigue cracking resistance of FAM mixtures. FTIR test results reveal that UV aging caused an increase in both the carbonyl and sulfoxide indices of asphalt binder. Furthermore, the carbonyl and sulfoxide indices in the 20% Buton rock asphalt modified asphalt binder increased by 52% and 50%, respectively, which were notably lower compared to those of the 70# virgin asphalt binder. These findings suggest that the inclusion of rock asphalt can significantly mitigate the impact of UV aging, thus potentially enhancing the aging resistance of FAM mixtures.

## Figures and Tables

**Table 1:** Asphalt binder types.

Binder ID	Asphalt Binder	Description
A	#70	#70 virgin asphalt binder
B	QC	#70 asphalt binder modified with 8% QC rock asphalt
C	UM	#70 asphalt binder modified with 8% UM rock asphalt
D	B8	#70 asphalt binder modified with 8% Buton rock asphalt
E	B20	#70 asphalt binder modified with 20% Buton rock asphalt
F	HMA	High-modulus binder from Xi’an Zhongli Asphalt Co., Ltd.

**Table 2:** The technical indices of asphalt binder.

Properties	Asphalt Binder						Standard
	#70	QC	UM	B8	B20	HMA	
Penetration (25 °C, 0.1 mm)	74.0	41.0	70.8	50.8	25.7	39.3	T 0604
Ductility (15 °C, cm)	>100	37.7	41.0	15.9	43.7	41.4	T 0605
Softening point (°C)	49.0	55.0	51.2	51.5	58.9	80.3	T 0605

**Table 3:** The properties of aggregate.

Aggregate Specification (mm)	Bulk Density (g/cm^3^)	Apparent Density (g/cm^3^)	Standard
0.6~1.18	2.670	2.711	T 0328-2005
0.3~0.6	2.665	2.697	
0.15~0.3	2.689	2.689	
0.075~0.15	2.704	2.704	
0~0.075	2.721	2.721	T 0353-2000

**Table 4:** The air void content of the specimens.

Asphalt Binder	Air Void Content (%)	Standard
#70 FAM mixture	0.39	T 0705
QC FAM mixture	0.42	T 0705
UM FAM mixture	0.35	T 0705
B8 FAM mixture	0.30	T 0705
B20 FAM mixture	0.48	T 0705
HMA FAM mixture	0.38	T 0705

## Data Availability

The original contributions presented in the study are included in the article, further inquiries can be directed to the corresponding author.
